# Shifting the Paradigm: The Transformative Role of Neoadjuvant Therapy in Early Breast Cancer

**DOI:** 10.3390/cancers16183236

**Published:** 2024-09-23

**Authors:** Nader Hirmas, Johannes Holtschmidt, Sibylle Loibl

**Affiliations:** 1 German Breast Group, 63263 Neu-Isenburg, Germany; nader.hirmas@gbg.de (N.H.); johannes.holtschmidt@gbg.de (J.H.); 2 Faculty of Medicine, Goethe University Frankfurt, 60590 Frankfurt, Germany

**Keywords:** neoadjuvant, breast cancer, pathological complete response, survival, biomarkers, efficacy, safety, HER2-positive, triple-negative breast cancer, hormone receptor-positive

## Abstract

**Simple Summary:**

Neoadjuvant therapy, used before surgery, is an important part of breast cancer treatment. Not only can it shrink tumors, but it can also provide valuable information on how the cancer responds to treatment, which can help physicians tailor further therapy. For aggressive types of breast cancer, such as HER2-positive and triple-negative breast cancers, targeted therapies and immunotherapy are often added to standard chemotherapy. Achieving a complete response to treatment (i.e., total absence of viable tumor from tissue removed during surgery) is usually a good sign, but it does not always mean better long-term outcomes for every patient. This review examines the evolution of neoadjuvant therapy in high-risk breast cancer and discusses recent clinical trials focused on optimizing treatment. It also highlights the need for new approaches to improve outcomes, especially for patients whose cancer does not fully respond to initial treatments.

**Abstract:**

The use of neoadjuvant systemic therapy (NST) has become increasingly important in the treatment of breast cancer because of its various advantages. These include the ability to downstage tumors without compromising locoregional control and the potential to obtain valuable information about clinical and biological response to therapy with implications for individual prognoses. Surgical response assessment paves the way for response-adapted therapy, and pathological complete response (pCR; defined as ypT0/is ypN0) serves as an additional endpoint for drug development trials. Recommended NST regimens commonly consist of anthracyclines and taxane, with dose-dense anthracyclines and weekly paclitaxel often preferred, whenever feasible. For patients with human epidermal growth factor receptor-2 (HER2)-positive tumors, dual anti-HER2 therapy (trastuzumab and pertuzumab) is indicated together with NST in case of elevated risk of recurrence. For patients with triple-negative breast cancer (TNBC), adding carboplatin to NST correlates with improved pCR and survival rates, as does the addition of immune checkpoint inhibitors. For hormone receptor (HR)-positive/HER2-negative cancers, emerging data on NST including immune checkpoint inhibitors may elevate the significance of NST in high-risk luminal breast cancer. Here, we present a synthesis of the results from neoadjuvant clinical trials that aim at optimizing treatment options for patients with high-risk breast cancer.

## 1. Introduction

The introduction of neoadjuvant systemic therapy (NST) in the treatment of breast cancer has been associated with various benefits. Initially, the most prominent benefits of NST were its ability to reduce tumor size and downstage locally advanced tumors, thereby increasing the likelihood of breast-conserving surgery (BCS) [1,2,3,4,5] and reducing postoperative complications without compromising survival outcomes [6,7]. Nowadays, and in addition to the above, NST allows clinicians to incorporate treatment response into treatment plans, and, accordingly, either reduce or enhance the intensity of locoregional and/or systemic adjuvant treatments. Response-adapted therapy can thus be used to avoid side effects from potentially unnecessary treatments. Patients whose tumors exhibit a clinical response to systemic therapy may be candidates for de-escalated treatment, e.g., replacement of complete axillary lymph node dissection (ALND) with targeted axillary dissection (TAD) or even sentinel lymph node biopsy [8,9,10,11,12,13], or the omission of adjuvant radiotherapy altogether [14]. On the other hand, more radical local therapies such as ALND and/or adjuvant radiotherapy as well as further chemotherapy may be more justified in patients whose tumors exhibit inadequate response to NST [15,16].

Data from the NSABP B-18 and NSABP B-27 trials [1,7] showed that outcomes in terms of disease-free survival (DFS) or overall survival (OS) in patients with early-stage breast cancer did not differ if the same chemotherapy was applied in the neoadjuvant and adjuvant setting. However, results showed that patients who achieved a pathological complete response (pCR) either defined as ypTis/ypN0 or ypT0/ypN0 had significantly better survival outcomes compared to those with residual disease post-NST. Similarly, an Early Breast Cancer Trialists’ Collaborative Group (EBCTCG) meta-analysis [17] showed that responders to NST had lower distant recurrence and lower breast cancer mortality than did non-responders. Further pooled analyses revealed that this association was strongest among patients with triple-negative breast cancer (TNBC) and those with human epidermal growth factor receptor-2 (HER2)-positive breast cancer [18,19]. Patients with hormone receptor (HR)-positive/HER2-negative breast cancer also benefit from NST; however, the difference between pCR and non-pCR achievement is smaller in comparison.

Hence, the presence of pCR and its association with survival endpoints have been used to develop treatment algorithms by assigning patients with residual disease and elevated risk of recurrence to further post-neoadjuvant treatments. So far, clinical implications have been seen with trastuzumab emtansine (T-DM1) in patients with HER2-positive disease and with capecitabine in patients with TNBC, where the prognostic value of pCR is known to be the highest [16,20,21].

In 2014, the U.S. Food and Drug Administration (FDA) accepted pCR as a surrogate marker for clinical efficacy in the neoadjuvant setting, with the condition that preliminary results must be confirmed by long-term survival data. Subsequently, pCR was incorporated in many clinical trials as a main endpoint [22,23]. This has led to the accelerated approval of pertuzumab as part of the neoadjuvant treatment of patients with HER2-positive breast cancer following results from the NeoSphere trial [24,25]. The results were later confirmed by the parallel running pure adjuvant APHINITY trial [26,27]. But this remains the only case in which a drug was approved based on a neoadjuvant study.

Not only has the introduction of NST contributed to improving outcomes for patients with high-risk breast cancer via more efficient treatment allocation, but it has also created the opportunity for integrating translational research to further understand the molecular causes of treatment failure. The neoadjuvant setting provides valuable information on tumor response, via tumor and blood samples collected before, during, and after therapy, hence facilitating the search for biomarkers predictive of survival outcomes. Baseline biopsies are commonly used to categorize patients according to tumor subtypes, which ultimately determines assignment to specific systemic treatments. Additional biopsies taken during treatment can identify early biomarkers of response (e.g., Ki-67 decrease after endocrine treatment in HR-positive breast cancer [28,29]), and residual tissues retrieved after NST could be used to identify biomarkers of resistance, which could potentially provide information about further treatment options and prognosis (e.g., the use of NanoString technology in TNBC identified the *DUSP4* gene—a negative regulator of ERK—a low expression of which is associated with greater tumor proliferation, worse survival, and enhanced sensitivity to MEK inhibition [30]).

Furthermore, in cases where patients need to undergo germline testing for *BRCA1/2* pathogenic variants [31], the neoadjuvant setting provides a buffer time, where therapy is provided until the results of genetic tests are available to be implemented into further treatment planning (including post-neoadjuvant therapy). In cases where the same adjuvant chemotherapy would be indicated, neoadjuvant chemotherapy (NACT) may be preferred, thereby yielding similar long-term outcomes [1]. Moreover, in settings with limited resources, in cases of public health crises (such as the recent SARS-CoV-2 pandemic) or in the event of an inability to undergo surgery, NST can provide disease control until surgery can be performed [32].

Of course, the effective implementation of NST algorithms in breast cancer treatment presents a number of considerable challenges in a real-world setting. These include the difficulty of identifying the most appropriate patients, ensuring the availability of essential resources such as advanced imaging, pathology, and newly approved drugs, as well as having alternative treatment strategies in place for patients who are unable to receive NST according to the trial setting. Consequently, the realization of more personalized treatment strategies requires broader access to diagnostic tools, multidisciplinary treatment planning, and more precise patient selection criteria to maximize the efficacy of NST and achieve superior outcomes across diverse healthcare settings. 

With regards to particular chemotherapy regimens, another EBCTCG meta-analysis compared long-term outcomes associated with different chemotherapy combination regimens for over 100,000 patients with early-stage breast cancer [33]. Results revealed that taxane/anthracycline-based regimens had a significantly improved outcome in comparison with anthracycline-based regimens (relative risk [RR] 0.86, *p* = 0.0005). Dose-dense doxorubicin and cyclophosphamide (AC) followed by paclitaxel, or dose-dense epirubicin and cyclophosphamide (EC) followed by docetaxel, have been extensively studied in clinical trials. These regimens have been shown to be effective in improving survival and pCR rates, and the sequential administration of taxane- and anthracycline-based therapy has been found to be superior to concomitant administration [34,35,36,37,38]. Such taxane/anthracycline-based regimens constitute an essential backbone of NACT in breast cancer, regardless of tumor subtype.

In this paper, we present a synthesis of the results from neoadjuvant clinical trials that guide optimum treatment options for patients with HER2-positive, TNBC, and HR-positive/HER2-negative breast cancer. While the first generation of studies investigated whether NACT is as good as adjuvant chemotherapy, the second generation tried to refine treatment regimens and improve pCR rates overall and in the individual subtypes.

## 2. Neoadjuvant Treatment of HER2-Positive Breast Cancer 

The prognosis of patients with HER2-positive breast cancer has greatly improved following the introduction of HER2-targeted therapy. Overall, patients with node-positive or high-risk node-negative HER2-positive breast cancer benefit from NACT combined with trastuzumab with or without pertuzumab (Table 1).

### 2.1. Trastuzumab ± Pertuzumab

Trastuzumab has been a gamechanger in the treatment and survival of patients with HER2-positive breast cancer. The NOAH study [44] revealed a 19% pCR increase when trastuzumab is added to NACT compared to NACT alone, and this translated to improved invasive DFS (iDFS) and OS. A pooled analysis by Petrelli et al. [58] revealed increases in pCR rates (20 to 43%) and decreases in relapse rates (20 to 12%) when trastuzumab is added to taxane/anthracycline-based NACT, and a pooled analysis by von Minckwitz et al. [59] showed a 3.2-fold increase in pCR odds in patients with HER2-positive breast cancer treated with trastuzumab during NACT (*p* < 0.001).

Furthermore, the addition of pertuzumab to trastuzumab significantly increases pCR rates in combination with NACT [60,61,62,63,64,65,66]. The NeoSphere trial [25] showed that docetaxel + dual blockade with pertuzumab and trastuzumab was associated with a significantly higher pCR rate compared to docetaxel + trastuzumab alone (45.8% vs. 29.0%; *p* = 0.0141). Patients who received chemotherapy-free neoadjuvant treatment had fewer serious adverse events compared to the other groups (4% vs. 10–17%), but at the cost of lower pCR rates. Following the pCR results from the NeoSphere trial, the FDA granted accelerated approval of pertuzumab in the neoadjuvant treatment of HER2-positive early breast cancer, which represented the first FDA approval of a specific neoadjuvant breast cancer treatment. 

Confirmatory trials to assess the survival benefit were demanded by the regulatory authorities for definitive approval. Survival outcomes of the NeoSphere trial were reported later in a descriptive fashion [24], since the study was not powered to detect differences in survival outcome. The combination of dual HER2-blockade and docetaxel showed numerically better disease-free survival (DFS) rates, but they were not statistically significant. The neoadjuvant TRYPHAENA trial [47,48] was also crucial in this context, since it assessed the cardiac safety of anthracycline-containing NACT compared to anthracycline-free NACT, each combined with trastuzumab and pertuzumab (i.e., ACTH-P vs. TCH-P). Even though no formal comparisons between the arms were made concerning efficacy, the trial reported an impressive pCR rate (ypT0/pTis) of 66.2% for TCH-P. The APHINITY trial was confirmatory in this regard, showing improved rates of iDFS with the addition of pertuzumab to trastuzumab and standard adjuvant chemotherapy [26,27].

These results were also corroborated with meta-analyses that confirmed statistically higher pCR rates with dual anti-HER2 blockade plus NACT compared to NACT alone, single anti-HER2 targeting with chemotherapy, or dual targeting without chemotherapy [60,63], and that the combination of trastuzumab and pertuzumab significantly improved pCR compared to trastuzumab alone [66]. On the other hand, results from various studies [41,49,53,63,67,68] investigating the benefit of lapatinib in the neoadjuvant setting do not support its use in this context.

As a result, American Society of Clinical Oncology (ASCO) guidelines [69] recommend that, for patients with node-positive or high-risk node-negative, HER2-positive disease, an anthracycline-based/non-anthracycline-based chemotherapy regimen plus trastuzumab should be used, with the option of adding pertuzumab to these regimens (ACTH-P or TCH-P). The absolute benefit from the addition of pertuzumab varies, and only patients with node-positive disease seem to benefit from the combination treatment [26]. A common fear, however, is that the combination of anthracyclines and anti-HER2 therapy may increase the risk of cardiac toxicity. Those concerns can be alleviated based on the results of the BERENICE [70] and TRYPHAENA [47,48] studies, which have shown that HER2-targeted therapy and anthracycline-containing regimens in the neoadjuvant setting result in cardiac and general safety profiles consistent with those of prior studies, with no substantially increased cardiac risks reported. Of note, the FDA has recently approved the fixed-dose combination (pertuzumab, trastuzumab, and hyaluronidase-zzxf) with NACT for patients with early or locally advanced, inflammatory HER2-positive breast cancer and for high-risk patients with early HER2-positive breast cancer [71].

It is essential to keep in mind that, after NACT and anti-HER2 therapy, pCR occurs more often in T1-T2 tumors, those with cN-negative status, as well as in tumors with HR-negative disease [72]. Higher tumor stage, nodal involvement, and HR-negative disease are independent prognostic factors for event-free survival (EFS) and OS for patients who do not achieve pCR. In contrast, among patients with pCR, only tumor stage and nodal involvement were prognostic for EFS, and this was further restricted to tumor stage concerning OS. This underscores the necessity to identify patients who may be at risk and would benefit from an intensified therapeutic approach.

*PIK3CA* mutations are associated with lower pCR rates in breast cancer, regardless of subtype (*PIK3CA* mutated: 23.0% vs. wild-type 38.8%, *p* < 0.0001), and this is particularly significant for HER2-positive subtypes (multivariate OR 0.43; 95% CI, 0.24–0.79; *p* = 0.006) after neoadjuvant taxane/anthracycline-based chemotherapy + anti-HER2 treatment, even if a dual HER2 blockade is given [73,74,75] (Table 2). More specifically, HR-positive/HER2-positive tumors with *PIK3CA* mutations have significantly lower pCR rates compared to wild-type (7.6% vs. 24.2%, respectively, *p* < 0.001), unlike HR-negative/HER2-positive tumors (27.2% vs. 36.4%, respectively, *p* = 0.125) [75]. Next-generation sequencing (NGS) can therefore be used to identify potential biomarkers for therapy resistance in breast cancer, including *PIK3CA*. In particular, gene sequencing can help identify specific hotspot mutations associated with worse pCR following NACT in HER2-positive breast cancer [76]. The ongoing GeparPiPPa trial (NCT05306041) aims to access pCR rates for patients with HR-positive/HER-positive breast cancer with *PIK3CA* mutations treated with neoadjuvant endocrine therapy (ET) + trastuzumab/pertuzumab ± PI3K inhibitor inavolisib.

### 2.2. Trastuzumab Emtansine (T-DM1)

As for the use of T-DM1 for HER2-positive breast cancer, the KRISTINE trial [45] showed that patients with operable stage II-III HER2-positive breast cancer who received TCH-P had higher pCR rates than those who received T-DM1 + pertuzumab (55.7% vs. 44.4%; *p* = 0.016). In contrast, the T-DM1 + pertuzumab arm was associated with fewer grade 3–4 adverse events (13% vs. 64%) and fewer serious adverse events (5% vs. 29%), as well as longer maintenance of patient-reported health-related quality of life and physical function. Despite this, early outcome results from the KRISTINE trial showed an increased risk of early progression in the experimental arm, which were attributed to heterogenous HER2 expression and a possible undertreatment of HER2-negative subclones in the T-DM1 + pertuzumab arm [46]. 

Unlike the neoadjuvant setting, however, the use of T-DM1 in the post-neoadjuvant setting has been considered practice changing. This was based on the results from the phase III KATHERINE trial that investigated the use of post-NACT T-DM1 vs. trastuzumab in patients with invasive residual disease after NACT + trastuzumab [20]. The 3-year iDFS was 88.3% vs. 77.0% in the TDM-1 vs. trastuzumab group (HR 0.50, 95% CI, 0.39–0.64; *p* < 0.001), with lower risk of distant recurrence in patients who received T-DM1 compared to those who received trastuzumab (HR 0.60, 95% CI, 0.45–0.79) at the cost of more serious adverse events in the T-DM1 group (12.7% vs. 8.1%), with more frequent severe (≥G3) adverse events (25.7% vs. 15.4%). The results of this trial have led to an approval of T-DM1 in this particular setting. Long-term follow-up confirmed an OS benefit in the intention-to-treat population [21]. Accordingly, adjuvant T-DM1 is recommended in patients with early-stage HER2-positive disease if residual invasive disease is present after NACT and HER2-targeted therapy [69], which represents an opportunity for treatment escalation for patients with an elevated risk of recurrence.

### 2.3. Therapy Optimization

Patients with stage 1 node-negative HER2-positive breast cancer are generally not considered candidates for neoadjuvant therapy [31]. Based on the results of the single-arm phase II Adjuvant Paclitaxel and Trastuzumab (APT) trial [83,84], such patients could potentially be spared from the effects of overtreatment and could be well treated with de-escalated adjuvant chemotherapy with paclitaxel + trastuzumab. Moreover, neoadjuvant treatment of patients with T1c N0 disease remains controversial, as no response assessment is possible due to the adjuvant treatment schedule. ASCO guidelines [69] recommend, depending on the clinical circumstances, that these patients could potentially be considered candidates for NACT. Additional benefit from knowledge on the individual response to NACT can be achieved, yet at the risk of overtreatment. 

Furthermore, patients with cT1 clinically node-negative HER2-positive breast cancer who receive upfront surgery may be upstaged based on intra-operative findings (e.g., pT2 pN1). In this context, they could have possibly derived benefit from a neoadjuvant approach, as there is evidence of treatment benefit with full adjuvant chemotherapy including pertuzumab according to the APHINITY trial [26]. There is an absolute improvement of 4.5% in iDFS with the addition of pertuzumab in this patient subgroup, which does not include a potential benefit from polychemotherapy as compared to paclitaxel single agent in the APT trial.

### 2.4. A Potential Role for Immune Checkpoint Inhibitors

It is postulated that combined treatment with HER2-blockade as well as anti-programmed death 1 (PD-1) and anti-programmed death ligand 1 (PD-L1) antibodies would potentially enhance anti-tumor effects further or even reverse drug insensitivity and drug resistance. To this end, a few phase II and III studies were conducted to evaluate the effect of combination therapy on pCR.

The single arm phase II KEYRICHED-1 trial (NCT03988036) assessed pCR rates in patients with HER2-enriched early breast cancer receiving four cycles of dual anti-HER2 blockade in combination with the immune checkpoint inhibitor (ICI) pembrolizumab. First results [85] reported a centrally confirmed pCR rate of 46% in the 43 patients of the per-protocol-population (38.5% for HR-positive/HER2-positive compared to 58.5% in HR-negative/HER2-positive tumors). Moreover, a recent biomarker analysis for pCR in this study [86] revealed potential clinical makers (such as tumor grade, estrogen and progesterone receptor status, and cT and cN status) as well as molecular biomarkers (gene expression, immunohistochemical markers, and stromal tumor-infiltrating lymphocytes [sTILs]) that are significantly associated with pCR. The results indicate that, in the future, patients could potentially be selected for chemotherapy-free regimens and achieve clinically meaningful pCR rates comparable to those obtained with longer, more toxic chemotherapy-containing regimens. However, full publication of the results is still awaited, and further validation in larger trials is warranted. 

In contrast, the phase III IMpassion050 study evaluated the addition of atezolizumab or placebo to neoadjuvant dose-dense AC–paclitaxel + pertuzumab/trastuzumab in patients with high-risk, HER2-positive early-stage breast cancer, including patients with PD-L1-positive breast cancer [87]. pCR rates were not significantly different overall (62.4% vs. 62.7% in the atezolizumab and placebo groups, respectively; 95% CI, −9.2–8.6; *p* = 0.9551) and in the subgroup of PD-L1-positive tumors (64.2% vs. 72.5%; 95% CI, −20.6–4.0; *p* = 0.1846). Overall, the safety profile was consistent with that of atezolizumab in other combination studies.

Similarly, the phase III APTneo Michelangelo trial (NCT03595592) [88] showed that the addition of atezolizumab to chemotherapy and trastuzumab + pertuzumab did not significantly increase the rate of pCR compared to placebo (57.8% vs. 52.0%, respectively; HR 1.33, 95% CI, 0.95–1.86; *p* = 0.091). An exploratory analysis showed that adding atezolizumab to neoadjuvant AC followed by trastuzumab + pertuzumab and carboplatin/paclitaxel (HPCT) led to a 9.9% significantly higher pCR rate compared to HPCT and atezolizumab (OR 1.58; 95% CI, 1.07–2.33; *p* = 0.022), regardless of HR and PD-L1 status. High sTILs (≥30%) and PD-L1-positive tumors had a higher likelihood of pCR in all arms.

Given these results, the addition of ICI to standard NACT with single or dual HER2-blockade is not yet warranted, and more data are required to assess efficacy and impact on survival in this patient population.

In summary, taxane/carboplatin-based or taxane/anthracycline-based NACT combined with trastuzumab and pertuzumab represent the current standard of care for patients with node-positive or high-risk node-negative HER2-positive breast cancer. In case of pCR, anti-HER2 therapy is completed with trastuzumab for patients with node-negative disease, and with trastuzumab + pertuzumab for patients with node-positive disease, each with a duration of up to 12 months. In case of invasive residual disease, completion of treatment with T-DM1 is recommended.

A growing field of interest focuses on the replacement of NACT polychemotherapy with an antibody–drug conjugate (ADC)-based treatment. The phase III, randomized DESTINY-Breast11 trial (NCT05113251) compares neoadjuvant trastuzumab deruxtecan (T-DXd) monotherapy vs. T-Dxd monotherapy followed by paclitaxel, trastuzumab, and pertuzumab vs. standard of care regimen AC followed by paclitaxel, trastuzumab, and pertuzumab in patients with high-risk HER2-positive early-stage breast cancer. With regards to post-neoadjuvant therapy, the phase III, randomized DESTINY-Breast05 trial (NCT04622319) compares T-DXd vs. T-DM1 as adjuvant therapy in patients with high-risk HER2-positive early breast cancer and residual disease after NACT. The study is fully recruited, and results are eagerly awaited.

## 3. Neoadjuvant Treatment of TNBC 

**Figure 1:**
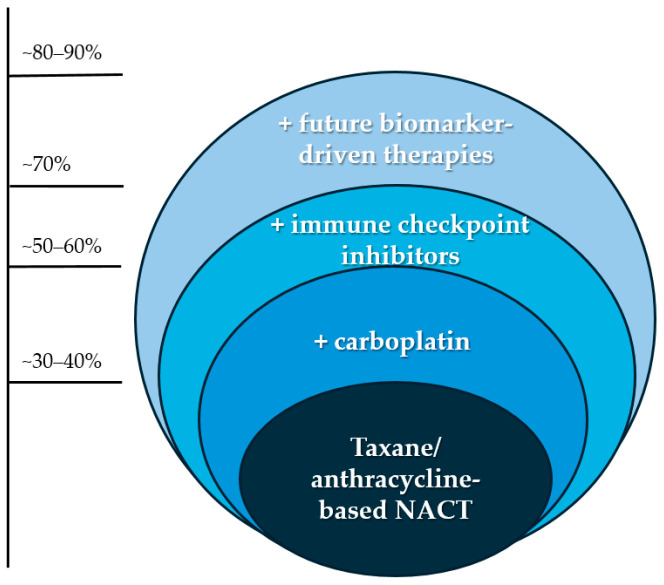
Increase in pCR rate in patients with TNBC by adding (+) carboplatin and immune checkpoint inhibitors. With more research into biomarkers and biomarker-driven therapies, pCR rates are expected to increase further.

While the majority of clinical trials with TNBC focused on increasing pCR rates by exchanging one taxane for another (e.g., GeparSepto and ETNA trials) or adding carboplatin to the treatment regimen (e.g., GeparSIixto, CALGB 40603, and BrighTNess trials), more recently, trials have shifted more towards the inclusion of ICI in the neoadjuvant setting. This has led to significant improvements in pCR rates and survival outcomes, and the results have been considered practice changing (Figure 1).

### 3.1. Chemotherapy Regimens

To begin with, studies have shown that chemotherapy regimens used in the adjuvant setting can also be appropriate in the neoadjuvant setting [17,89]. As such, current ASCO guidelines recommend that patients with clinically node-positive and/or at least cT1c TNBC be offered a taxane/anthracycline-based NACT regimen [69], which has been associated with improved outcomes [17]. Moreover, dose-dense AC followed by paclitaxel or dose-dense EC followed by docetaxel have shown an improvement in survival as well as an achievement of pCR, with dose-done anthracycline and weekly paclitaxel being the preferred regimen [34,35,36,37,38,90]. However, further research is needed to fully understand the optimal dosing and sequencing of these regimens in the neoadjuvant treatment of TNBC.

The GeparSepto trial showed that weekly nab-paclitaxel, in comparison to paclitaxel, followed by EC in the neoadjuvant setting contributes to a higher pCR rate (38% vs. 29%; OR 1.53, 95% CI, 1.20–1.95; unadjusted *p* < 0.001) and improved iDFS (84.0% vs. 76.3%; HR 0.66, 95% CI, 0.51–0.86; *p* = 0.002) in patients with early breast cancer [51,52]. However, the improved pCR with nab-paclitaxel vs. paclitaxel reported in the ETNA trial failed to reach statistical significance (22.5% vs. 18.6%, respectively; OR 0.77, 95% CI, 0.52–1.13; *p* = 0.19) [91], which was also true for the 5-year EFS (75.6% vs. 68.1%, respectively; HR 0.83, 95% CI, 0.60–1.14; *p* = 0.245) [92]. Nab-paclitaxel so far is only approved for the treatment of metastatic breast cancer. Nevertheless, it is frequently used off-label in the neoadjuvant treatment of distinct cases of TNBC, as well as a substitute in case of hypersensitivity reactions to solvent-based paclitaxel, and it has been used in several early immunotherapy trials [93,94]. The combination of nab-paclitaxel and carboplatin has been investigated; even though the continuous application seemed to lead to a higher pCR rate in TNBC and a trend for an improved iDFS, the toxicity, especially neurotoxicity, also increased. As a result, this regimen remains investigational [95,96]. 

As for the addition of platinum to standard anthracycline-based NACT, several phase II and III clinical trials have shown an improvement in pCR rates [97,98,99], yet there is an increase in the incidence of adverse events, as expected, and the effects on long-term survival outcomes have not been fully elucidated. In particular, GeparSixto [100] and BrighTNess [101] trials revealed an increase in EFS with the addition of carboplatin, but the CALGB 40603 study [102] did not reveal the same, yet the latter was not powered to evaluate EFS and still showed that patients who achieved a pCR had better survival outcomes compared to those who did not, even those with RCB class I. Most recently, Gupta et al. [103] showed that the benefit of carboplatin was confined to patients younger than 50 years of age, with 5-year DFS of 74.5% vs. 62.3% (*p* = 0.003) and 5-year OS of 76.8% vs. 65.7% (*p* = 0.004) with and without carboplatin, respectively. Full publication of the results is awaited to better understand the impact of this trial.

Furthermore, an intervention review by the Cochrane Breast Cancer Group [104] confirmed that platinum chemotherapy increases pCR rates (risk ratio (RR) 1.44, 95% CI, 1.31–1.59) as evident from a pooled analysis from 15 studies involving 3083 patients. Subgroup analyses showed no evidence of differences in DFS according to *BRCA* mutation status, homologous recombination deficiency (HRD) status, or lymph node status. The review also pointed to the fact that patients receiving platinum chemotherapy were more likely to require treatment delays (RR 2.23, 95% CI, 1.70–2.94), dose reductions (RR 1.77, 95% CI, 1.56–2.02), and early cessation of treatment (RR 1.20, 95% CI, 1.04–1.38). Increased hematological toxicity occurred in the platinum group, where patients were more likely to experience grade III/IV neutropenia (RR 1.53, 95% CI, 1.43–1.63), anemia (RR 8.20, 95% CI, 5.66–11.89), and thrombocytopenia (RR 7.59, 95% CI, 5.10–11.29). As a result, European Society for Medical Oncology (ESMO) and ASCO guidelines recommend adding carboplatin to the NACT regimen after considering benefits and risks for individual patients [31,69].

In the renowned CTNeoBC pooled analysis [18], a strong association between pCR and long-term survival in TNBC was documented (EFS: HR 0.24, 95% CI, 0.18–0.33; and OS: HR 0.16, 95% CI, 0.11–0.25). This brought up the clinical necessity of alternative therapy options for patients with TNBC without a pCR who are at a high risk of recurrence. Prior to incorporating nab-paclitaxel or carboplatin into neoadjuvant treatment schedules, the phase III CREATE-X trial had evaluated capecitabine as a treatment option for patients with HER2-negative tumors and residual disease post taxane/anthracycline-based NACT [16]. Standard dosed capecitabine for 6–8 cycles as a post-NACT treatment compared to control displayed pronounced survival effects in patients with TNBC with regards to DFS (69.8% vs. 56.1%; HR 0.58, 95% CI, 0.39–0.87) and OS rates (78.8% vs. 70.3%; HR 0.52, 95% CI, 0.30–0.90), respectively. However, the GEICAM/2003-11_CIBOMA/2004-01 trial [105] failed to show a statistically significant increase in DFS with extended capecitabine added to standard chemotherapy in patients with early TNBC. In a preplanned subset analysis, patients with a non-basal phenotype seemed to obtain benefit with capecitabine. In addition, the ECOG-ACRIN EA1131 trial [106] showed that, compared to post-neoadjuvant capecitabine, platinum did not improve outcomes in patients with basal subtype TNBC with residual disease post-NACT, and it was associated with more severe toxicity.

### 3.2. Immunotherapy Regimens

Recently, immunotherapy has emerged as a promising therapeutic avenue for TNBC, particularly through the inhibition of immune checkpoint pathways. ICI, such as PD-1/PD-L1 inhibitors, aim to enhance the immune response against cancer cells by blocking inhibitory signals that allow tumors to evade immune detection [107]. It is postulated that treatment with ICI and NACT enhances the immune response via the release of neoantigens as well as a more improved adaptive immune response compared to treatment with these agents after the primary tumor is resected [108].

Table 3 lists the major clinical trials that have investigated the efficacy of immunotherapy in the neoadjuvant setting for early-stage TNBC. The addition of ICI to standard chemotherapy regimens has shown improvements in pCR rates, but the side effect profile varies across patients, ranging from acceptable to chronic and even life-threatening [109].

The pivotal placebo-controlled and randomized phase III KEYNOTE-522 trial investigated the addition of PD-1 inhibitor pembrolizumab to anthracycline/taxane-based NACT including carboplatin, followed by pembrolizumab or placebo after surgery for stage II/III early TNBC [110]. The trial reported a significant increase in pCR, and later on, there was a significant improvement in EFS [111] (Table 3). The results have been considered practice changing. An improvement was seen across PD-L1 positive and negative subgroups in the neoadjuvant setting, unlike in the metastatic setting, where survival outcomes improved among patients with TNBC and PD-L1 positive disease only [115]. OS data from the trial are eagerly awaited.

Nevertheless, uncertainty persists regarding the optimal approach for patients who do not achieve a pCR and the optimal duration of ICI—irrespective of pCR—as a component of further adjuvant therapy. For example, in the neoadjuvant GeparNuevo trial comparing PD-L1 inhibitor durvalumab to placebo given with NACT, a moderate increase in pCR of 9.2% translated into an improved iDFS, DDFS, and OS, even though the schedule did not include adjuvant continuation of ICI therapy [93]. On the other hand, the IMpassion031 trial comparing PD-L1 inhibitor atezolizumab to placebo given with nab-paclitaxel-EC followed by atezolizumab or placebo after surgery did show a significant improvement in pCR, but it demonstrated only a nonsignificant improvement in EFS with a strong trend for OS [94,116]. The ALEXANDRA/IMpassion030 trial investigating the addition of atezolizumab to classical adjuvant chemotherapy has recently reported final negative results [117], prompting speculations whether the presence of relevant primary tumor is necessary to provoke an adequate immune response, as this had been shown preclinically [118] and clinically for melanoma [119]. Similarly, the A-BRAVE trial failed to show an improvement in DFS with adjuvant avelumab vs. observation for patients with early TNBC and residual disease post-NACT or at high risk after primary surgery and adjuvant chemotherapy [120]. Nevertheless, OS was significantly improved for the total population.

Post-neoadjuvant ICI therapy remains controversial, given that both capecitabine [16] and olaparib (for germline *BRCA*-associated breast cancer) [15] are recommended post NACT treatments for patients with TNBC and residual disease according to the initial trials with a documented OS benefit [15,16]. The phase III SACIA trial (GBG 102, NCT04595565) is ongoing to evaluate post-NACT therapy with sacituzumab govitecan (SG), an ADC that has proven to be effective in metastatic breast cancer previously treated with taxane/anthracycline-based chemotherapy, in comparison to control (treatment of physician’s choice). The importance of a possible combination of ICI with post-NACT capecitabine or ADC therapy will have to be extrapolated from the recently initiated phase III ASCENT-05/OptimICE-RD (AFT-65, GBG 119, NCT05633654) comparing SG + pembrolizumab vs. pembrolizumab ± capecitabine (per treating physician’s discretion) in patients with TNBC and residual disease post NACT. Several other trials are currently investigating the role of an ADC with and without ICI In the neoadjuvant and post-neoadjuvant setting (e.g., NeoSTAR (NCT04230109), I-SPY2 (NCT01042379), TROPION-Breast03 (NCT05629585), and TROPION-Breast04 (NCT06112379)). Furthermore, a definitive answer to the question of whether adjuvant ICI therapy is necessary in patients with pCR is anticipated with non-inferiority trials like the currently enrolling phase III OptimICE-PCR (NCT05812807), which compares the effect of pembrolizumab to observation for patients with early-stage TNBC who achieved a pCR after NACT that included pembrolizumab. In summary, further research is warranted to elucidate the impact of adjuvant immunotherapy on long-term survival, and to identify predictive biomarkers for treatment response.

Currently, biomarkers are investigated in the post-neoadjuvant setting to detect minimal residual disease (MRD) post-op to help further guide treatment decisions. For example, in patients with TNBC and MRD post NACT, detection of circulating tumor DNA (ctDNA) has been significantly associated with lower DFS and OS [81,121] (Table 2). As such, ctDNA detection is currently being studied to tailor immunotherapy after completion of primary TNBC treatment. For example, the phase II c-TRAK-TN trial (NCT03145961) involves a serial ctDNA surveillance component as well as a therapeutic component with pembrolizumab for one year in the case of ctDNA detection, to assess the ability of ctDNA to detect cancer cells after standard treatment in addition to pembrolizumab in early-stage TNBC. 

Increased TILs have also been investigated as biomarkers to guide further treatment plans. For example, one way to distinguish between patients with stage I TNBC who would require adjuvant therapy and those with low risk of recurrence for whom additional treatment is not beneficial is via increased TILs [122,123]. Moreover, it has been shown in the GeparNuevo trial that an increase in sTILs predicts higher pCR rates overall [113], and a recent meta-analysis showed that this translates to significant improvements in DFS and OS [78] (Table 2). Using whole exome sequencing and RNA-sequencing data from the same study, it was shown that tumor mutational burden as well as immune gene expression profiles or TILs are independent predictors for pCR as well [124]. This calls for further analyses of tumor mutational burden in combination with immune parameters to tailor therapies in TNBC.

### 3.3. PARP Inhibitor Regimens and BRCA1/2 Mutation Testing

The OlympiA trial evaluated the effect of the poly(ADP-ribose) polymerase inhibitor (PARPi) olaparib compared to placebo for one year as a post-NACT treatment in patients with clinicopathological high-risk HER2-negative early breast cancer and germline pathogenic or likely pathogenic *BRCA1* or *BRCA2* variants [15]. Patients with TNBC could be included if they had a high clinical risk after adjuvant chemotherapy or if they had residual invasive disease following NACT. The trial reported a significant improvement in OS after a median of 3.5 years (HR 0.68; 98.5% CI 0.47–0.97, *p* = 0.009). Subset analyses for survival parameters displayed benefits across major subgroups [125].

In addition, the phase II non-randomized open-label NEOTALA trial assessed the efficacy of talazoparib in patients with germline *BRCA1/2* mutated, HER2-negative breast cancer [126]. Patients were given neoadjuvant talazoparib for 24 weeks followed by surgery. For the 48 evaluable patients, all of them had TNBC, and a pCR rate of 45.8% was reported (49.2% in the intention-to-treat population). This early-phase trial could represent an exciting non-chemotherapy treatment option, but larger confirmatory clinical trials with long-terms outcomes are needed. A follow-up biomarker analysis of the trial [127] revealed a strong concordance (97.8%) between tumor and germline *BRCA* mutations and high prevalence (98%) of *TP53* mutations. This highlights the central role of *BRCA* and *TP53* mutations in this context and supports the assessment of germline *BRCA* status for the molecular eligibility of talazoparib in patients with TNBC.

Furthermore, *BRCA1/2* mutation status can also be used to predict treatment outcomes. In the GeparOcto retrospective biomarker study [82], higher pCR rates were observed in patients with *BRCA1/2* variants than in patients without (60.4% vs. 46.7%; odds ratio [OR], 1.74; 95% CI, 1.13–2.68; *p* = 0.01). In contrast, variants in non-*BRCA1/2* breast cancer predisposition genes were not associated with therapy response (Table 2). This is particularly true for patients with TNBC and HR-positive/HER2-negative breast cancer, which suggests that germline *BRCA1/2* testing should be considered prior to treatment start.

## 4. Neoadjuvant Treatment of HR-Positive/HER2-Negative Breast Cancer

In HR-positive breast cancer, pCR rates after NACT are known to be comparably low, especially in low- and middle-grade tumors, ranging from 7% for low-grade tumors to 16% for high-grade ones [18]. However, the prognostication ability for pCR differs depending on the breast cancer subtype; the ability for pCR to predict DFS was not impactful in luminal A, but rather in luminal B/HER2-negative breast cancer [128]. Hence, patients with luminal A disease should not be given NACT, unless absolutely necessary (such as in the case of locally advanced or inoperable disease, for instance). In contrast, luminal B tumors have a higher likelihood of response; hence, it is necessary to consider other variables, such as histology, tumor size, and initially involved lymph nodes, in addition to pCR, when evaluating the risk of relapse. High-risk patients with luminal B tumors could still potentially benefit from additional treatment in the post-neoadjuvant setting [129]. 

### 4.1. Chemotherapy and Endocrine Therapy

With combined taxane/anthracycline-based NACT as a suitable option, additional treatments have been evaluated for the neoadjuvant treatment of HR-positive/HER2-negative breast cancer. Non-anthracycline-based regimens may also be used in the neoadjuvant settings based on various data from the adjuvant setting [130,131]. Unlike in TNBC—as discussed earlier—the addition of carboplatin for patients with HR-positive/HER2-negative breast cancer results in more toxicities and does not significantly impact the rate of pCR or EFS [132]; hence, it is not a suitable option for this tumor subtype. As such, the chemotherapy regimen of choice in high-risk HR-positive/HER2-negative breast cancer remains taxane/anthracycline-based treatment, with TC being a valid option in patients incapable of receiving anthracyclines.

With regards to the use of ET vs. NACT, a meta-analysis by Spring et al. [133] involving 20 clinical trials and 3490 patients showed that, in postmenopausal women, neoadjuvant aromatase inhibitors (AIs) were more effective than tamoxifen and comparable to combination NACT in terms of improved clinical response and BCS rates. In addition, and as expected, toxicity is significantly greater with NACT in comparison to ET. Moreover, ET monotherapy vs. dual ET showed no difference in clinical response rates. Hence, in this patient population, ET monotherapy with Ais appears to be an efficacious treatment option. Furthermore, neoadjuvant approaches combining ET and cyclin-dependent kinase (CDK)4/6 inhibitors yielded enhanced clinical response rates [134,135,136,137], yet the evidence is still premature given that such studies are small phase II and proof-of-concept studies.

As such, ASCO guidelines [69] recommend considering neoadjuvant ET in larger tumors for which tumor downstaging is desired. But one must expect that a pCR in this case is rare, and the use of neoadjuvant ET is frequently triggered by a temporary or ongoing incapacity to receive surgery. ASCO guidelines [69] recommend that the optimal duration of neoadjuvant ET should be individualized and guided by careful evaluation of the patient’s clinical status and the clinical response over time. Most studies that reported downstaging of tumors with neoadjuvant ET administered 3–6 months of treatment, which is currently recommended.

For premenopausal women, on the other hand, neoadjuvant ET has been less rigorously investigated. The STAGE study [138] evaluated neoadjuvant tamoxifen plus goserelin vs. anastrozole plus goserelin in premenopausal women with estrogen receptor (ER)-positive, early-stage breast cancer. Clinical complete and partial responses were seen in 70.4% vs. 50.5% of patients in the anastrozole/goserelin and tamoxifen/goserelin groups, respectively (19.9% difference, 95% CI, 6.5–33.3; *p* = 0.004). Existing data indicate that neoadjuvant ET is likely less effective than chemotherapy if downstaging is desired. A study by Kim et al. [139] confirmed that NACT yields higher response rates than neoadjuvant ET at the cost of higher toxicity. 

In case of clinical indecisiveness in individual patients, ASCO-endorsed biomarker assays, such as Oncotype DX, MammaPrint, Breast Cancer Index (BCI), and EndoPredict [140] can assist treatment decision making regarding the use of (neo-)adjuvant chemotherapy. Retrospective studies showed that a higher Oncotype DX risk score (RS) correlates with higher pCR rates compared to a lower RS [141], while a lower RS indicates that neoadjuvant ET may be a better approach for some patients [142]. Another study showed that EndoPredict molecular/clinical low risk patients are probably sufficiently treated with standard neoadjuvant chemotherapy followed by ET, whereas those with high-risk scores have an increased risk of recurrence despite such therapy [143], where the latter would be suitable candidates for novel therapy approaches, for example.

Other prognostic signature scores provide similar results in terms of high scores predicting response to chemotherapy in the neoadjuvant setting, likely reflective of more proliferative tumors [144]. For instance, studies have shown the utility of Ki67 after short-term preoperative ET in predicting recurrence-free survival and (indirect) response to adjuvant therapy [28,145,146]. Therefore, in cases where gene expression assays are not particularly informative or meaningful, endocrine responsiveness assessed by Ki67 reassessment after short NET can be used to identify patients who would potentially not benefit from adjuvant chemotherapy. 

However, the role of prognostic signature scores in guiding neoadjuvant therapy remains uncertain and has not yet been fully adopted in clinical practice. Further studies are underway to determine the clinical utility of multi-gene prognostic signature scores in the neoadjuvant setting (e.g., PLATO study (NCT03900637) and NACAGEP study (NCT05666258)).

### 4.2. Adaptive Therapy

Novel clinical trial designs in the neoadjuvant setting for this patient population are shifting course towards assessing endocrine responsiveness and adapting treatment accordingly. For example, the POETIC trial from the UK [29] investigated the use of neoadjuvant AI therapy in postmenopausal patients with operable HR-positive breast cancer. Patients either received or did not receive letrozole or anastrozole pre- and post-surgery followed by standard adjuvant treatment. Ki67 (dichotomized at 10%) as a predictive biomarker was measured at baseline and at 14 days post treatment to assess its predictive ability. While the study failed to show improvements in long-term benefits with such treatment, it has provided evidence for the clinical validity of Ki67 as a prognostic biomarker (baseline and at a 2-week timepoint) to identify patients with higher risk of recurrence despite standard-of-care ET therapy. Patients with low baseline Ki67 or with a response to short-term ET at 2 weeks are not likely to benefit from adjuvant chemotherapy, while those whose 2-week Ki67 remains high or does not decrease to at least below 10% might benefit from further adjuvant chemotherapy or innovative therapies beyond ET monotherapy.

Similarly, in Germany, the WSG ADAPT trial program [147] seeks to investigate early response assessment and subtype-specific therapy tailoring to patients with the highest likelihood of benefit. The ongoing WSG-ADAPTcycle trial is a prospective, multi-center, open-label, phase III trial (NCT04055493) that aims to compare outcomes of patients with HR+/HER2− early breast cancer and intermediate risk if treated with 2 years of the CDK4/6 inhibitor ribociclib combined with ET compared to chemotherapy, each followed by standard ET. Results from the neoadjuvant ET screening window phase showed that endocrine response is dependent on the type of ET, age, and genomic risk as assessed by Oncotype DX [148].

### 4.3. Immunotherapy

Compared to TNBC, HR-positive/HER2-negative breast cancer is less immunogenic and has frequently less TILs and a lower tumor mutational burden [108]. However, some high-risk ER-positive/HER2-negative breast cancers also have high TIL counts and immune gene expression levels similar to those seen in TNBC [149], with potential to benefit from ICI therapy. Interestingly, while an increase in TILs was associated with longer DFS in TNBC and HER-positive breast cancer, as well as longer OS in TNBC, it was not associated with longer DFS in luminal HER2-negative tumors, and OS was in fact shorter [150].

Although the rationale is weak to employ ICI in early HR+/HER2− breast cancer therapy, two placebo-controlled phase III trials have assessed the addition of ICI to NACT in high-risk, stage II-III, ER-positive/HER2-negative breast cancer and reported pCR and biomarker results. These are the KEYNOTE-756 trial [151] (NCT03725059) with pembrolizumab and the CheckMate 7FL trial (NCT04109066) [152] with nivolumab as the ICI of choice. The KEYNOTE-756 trial showed a statistically significant improvement in pCR rate in the pembrolizumab arm compared to placebo (24.3% vs. 15.6%; *p* = 0.00005), and a similar trend was seen in the Checkmate 7FL trial in the nivolumab arm (24.5% vs. 13.8%; *p* = 0.0021) and in the stage III subgroup (Arm A/B, 30.7%/8.1%). 

In both studies, the benefit of an ICI on pCR was higher in patients whose tumors were positive for PD-L1 expression (29.7% versus 19.6% for pembrolizumab vs. placebo, respectively; and 44.3% vs. 20.2% for nivolumab vs. placebo, respectively). Moreover, in the CheckMate 7FL trial, residual cancer burden (RCB) 0–1 rates improved overall and more so in patients with PD-L1-positive tumors. In addition, patients whose tumors had sTILs > 1% derived more benefit with the addition of nivolumab. However, it is worth noting that the discordance in PD-L1 positive proportions between KEYNOTE-756 vs. CheckMate-7FL (76% vs. 35%) is due to the different scoring methods and assay sensitivities [153]. Due to such variations, PD-L1 expression may not be optimal to identify patients with ER-positive/HER2-negative tumors who need more effective chemotherapy plus ICI to improve survival. 

Furthermore, the I-SPY2 trial [114] examined the addition of pembrolizumab to NACT for patients with HER2-negative breast cancer. In patients with HR-positive/HER2-negative breast cancer considered high-risk using MammaPrint gene expression assay, the addition of pembrolizumab increased the pCR rate from 13% to 30%. Further molecular analysis revealed that among these cancers, only the MammaPrint High-2 (or MP2) subset exhibited an enhanced pCR rate. Given that MammaPrint has the potential to identify patients with ER-positive/HER2-negative cancers who may benefit from adjuvant therapy, the MP2/High-2 classification could effectively delineate the subpopulation among the chemotherapy-eligible patients who require immune checkpoint therapy [153]. 

The ongoing phase III SWOG S2206 trial (NCT06058377) evaluates this particular strategy and assigns patients with ER-positive/HER2-negative and MP2/High-2 cancers to durvalumab plus NACT vs. NACT alone. This study does not include adjuvant ICI, thereby allowing for a more rigorous evaluation of the impact of pCR rate improvement on recurrence-free survival.

It is crucial to acknowledge that the promising improvements in pCR rates observed with ICIs in the aforementioned trials require further follow-up to fully assess their impact on long-term outcomes, such as DFS and OS. This is because the correlation between pCR and treatment efficacy is less pronounced in HR-positive disease.

## 5. pCR: Challenges and Alternatives

The reliability of pCR as a predictor of prognosis varies depending on breast cancer subtypes and subsequent treatments received after the timepoint of pCR assessment. While some trials demonstrated a positive correlation between pCR and improved survival outcomes (e.g., NOAH and KEYNOTE-522), this correlation was not substantiated in other trials (e.g., IMpassion031). Additionally, other trials revealed significant survival gains that were not contingent on pCR (e.g., GeparNuevo). The reasons for this discrepancy are likely to be found in the disease subtype itself, the therapy applied, or alternatively, in the design of the trial. In particular, it can prove challenging to ascertain OS in neoadjuvant trials that enroll patients with ER-positive breast cancer. It is possible that patients may have experienced a relapse, but the advent of advanced metastatic breast cancer treatments has led to improved survival outcomes in such cases. Consequently, the occurrence of an OS event may fall within the time interval following the conclusion of the trial follow-up period. 

Moreover, the survival benefit derived by patients who exhibited a partial response but not a pCR is not accounted for, which could potentially compromise the reliability of pCR. The meta-analysis by Cortazar et al. [18], which pooled data from 12 neoadjuvant trials, revealed that while pCR was associated with improved survival at the individual level, it did not validate pCR as a reliable surrogate endpoint for enhanced iDFS or OS at the trial level. Hence, the use of pCR as a surrogate marker for survival remains complex and should be interpreted with caution, especially in the absence of long-term follow-up data to verify early reported improvements.

In recent years, other factors have emerged as important prognostic indicators beyond pCR. The RCB score has gained prominence as a more nuanced tool for predicting outcomes and guiding subsequent therapy decisions in breast cancer trials where pCR is not achieved [154]. The RCB score considers not only the size of the residual tumor (taking into account the proportion of in situ components) and cancer present in the lymph nodes, but also factors such as residual cancer cellularity, which has been previously reported to be a significant prognostic factor after NACT [155]. This provides a more comprehensive measure of residual disease burden than would be possible with a single factor. The I-SPY2 trial, for example, demonstrated that patients with lower RCB scores exhibited superior EFS compared to those with higher RCB scores, irrespective of subtype and treatment [156]. The implementation of effective NACT may result in a shift in the distribution of RCB scores within the experimental arm of a trial population towards lower scores, which are associated with a more favorable prognosis. These findings highlight the potential of RCB scores to offer more nuanced insights into patient prognosis and inform decisions regarding adjuvant therapy, even in instances where pCR is not achieved.

Furthermore, ctDNA has emerged as a promising biomarker with significant prognostic potential. The presence of ctDNA at baseline has been demonstrated to be associated with worse survival outcomes, and this correlation becomes even stronger when ctDNA is detected after NST and further intensifies when detected during the follow-up period [80,157] (Table 2). This highlights the importance of monitoring ctDNA dynamics throughout the course of treatment. Moreover, evidence indicates that ctDNA levels at the outset of NST are predictive of local tumor response upon completion of therapy [158], suggesting that early detection of ctDNA during NACT could facilitate real-time assessment of treatment efficacy and identification of patients who may not achieve optimal responses. As a result, the possibility of early escalation of treatment in high-risk patients, rather than waiting until the end of therapy, is a potential avenue for further investigation. This reinforces the need for more research into the role of ctDNA as a dynamic, real-time biomarker with the potential to improve outcomes by tailoring therapies according to individual risk profiles earlier in the treatment process.

In conclusion, as discussed so far and illustrated in Table 2, biomarkers such as *PIK3CA* mutational status, *BRCA1/2* mutations, TILs, and ctDNA have demonstrated considerable promise in predicting therapeutic responses and survival outcomes, particularly in TNBC and HER2-positive breast cancers. Nevertheless, while these markers hold potential, their utility has not yet been fully established across diverse breast cancer subtypes or broader patient populations. Further validation in larger, multicenter prospective trials is essential to ensure their applicability and reliability in clinical practice. By rigorously testing these biomarkers in varied settings and patient groups, we can move closer to personalized treatment strategies that more effectively target the unique biology of each patient’s cancer.

## 6. Conclusions and Future Directions

The neoadjuvant setting provides numerous opportunities for more efficacious therapies and the gathering of valuable insights from a translational perspective. Although achieving a pCR on an individual patient basis is associated with a favorable prognosis, evaluating pCR rates as a surrogate for improved survival on a trial level may not be the most optimal approach. Consequently, further prognostic markers are being investigated, including RCB, TILs, ctDNA analyses, as well as biomarker assays, which provide more comprehensive risk stratification and could potentially inform treatment decisions beyond pCR.

The utility of these biomarkers after NST must be validated through larger, prospective clinical trials. This will enable the integration of individual results on molecular residual disease into further adjuvant treatment plans and will also ensure their applicability across diverse populations and settings. Enhancing the reliability of these biomarkers has the potential to facilitate the development of more effective, personalized treatment approaches, which could also improve long-term outcomes. Such validation is critical for enhancing the resolution of treatment response and facilitating the refinement of escalation and de-escalation treatment strategies.

## Figures and Tables

**Table 1:** Clinical trials investigating the efficacy of HER2-targetted immunotherapy in the neoadjuvant setting for node-positive or high-risk node-negative HER2-positive disease.

Trial	N	Interventions	pCR % (95% CI)	Other Results (95% CI)
CALGB 40601 [39]	117	Paclitaxel, trastuzumab, and lapatinib	56% (47–65%)	No effect of dual HER2 therapy in the HR-positive subset, but a significant increase in pCR with dual therapy in HR-negative disease (*p* = 0.01). pCR rates differed by subtype (HER2 enriched, 70%; luminal A, 34%; luminal B, 36%; *p* = 0.001)
	118	Paclitaxel and trastuzumab	46% (37–55%)	
	64	Paclitaxel and lapatinib	32% (22–45%)	
Z1041 [40]	138	FEC-75 followed by paclitaxel and trastuzumab (sequential group)	56.5% (47.8–64.9%)	
	142	Paclitaxel and trastuzumab followed by FEC-75 (concurrent group)	54.2% (45.7–62.6%)	
NeoALTTO [41,42,43]	152	Lapatinib plus trastuzumab	51.3% (43.1–59.5%)	3-year EFS: 84% (77–89%) 3-year OS: 95% (90–98%)
	149	Trastuzumab	29.5% (22.4–37.5%)	3-year EFS: 76% (68–82%) 3-year OS: 90% (84–94%)
	154	Lapatinib	24.7% (18.1–32.3%)	3-year EFS: 78% (70–84%) 3-year OS: 93% (87–96%)
NeoSphere [24,25]	107	Trastuzumab + docetaxel	29.0% (20.6–38.5%)	5-year DFS: 81% (72–88%)
	107	Pertuzumab and trastuzumab + docetaxel	45.8% (36.1–55.7%)	5-year DFS: 84% (72–91%)
	107	Pertuzumab + trastuzumab	16.8% (10.3–25.3%)	5-year DFS: 80% (70–86%)
	96	Pertuzumab + docetaxel	24.0% (15.8–33.7%)	5-year DFS: 75% (64–83%)
NOAH [44]	117	Trastuzumab + AT + CMF	43% (breast), 38% (breast and ALNs)	5-year OS: 74% (64–81%) 5-year EFS: 58% (48–66%)
	118	AT + CMF	22% (breast), 19% (breast and ALNs)	5-year OS: 63% (53–71%) 5-year EFS: 43% (34–52%)
KRISTINE [45,46]	223	Trastuzumab + pertuzumab	44.4%	3-year EFS: 85.3% (80.5–90.1%) 3-year iDFS: 93.0% (89.4–96.7%)
	221	Docetaxel, carboplatin, and trastuzumab + pertuzumab	55.7%	3-year EFS: 94.2% (91.0–97.4%) 3-year iDFS: 92.0% (86.7–97.3%)
TRYPHAENA [47,48]	73	FEC with concurrent trastuzumab + pertuzumab, then docetaxel with trastuzumab + pertuzumab	61.6%	3-year DFS: 87% (79–95%) 3-year PFS: 89% (81–96%)
	75	FEC followed by docetaxel with trastuzumab + pertuzumab	57.3%	3-year DFS: 88% (80–96%) 3-year PFS: 89% (81–96%)
	77	Docetaxel, carboplatin, and trastuzumab + pertuzumab	66.2%	3-year DFS: 90% (82–97%) 3-year PFS: 87% (80–95%)
NSABP B-41 [49]	174	AC followed by paclitaxel and trastuzumab plus lapatinib	62.0% (54.3–68.8%)	pCR in breast and negative nodes: 60.2% (52.5–67.1%)
	174	AC followed by paclitaxel and lapatinib	53.2% (44.9–59.5%)	pCR in breast and negative nodes: 47.4% (39.8–54.6%)
	181	AC followed by paclitaxel and trastuzumab	52.5% (44.9–59.5%)	pCR in breast and negative nodes: 49.4% (41.8–56.5%)
ABCSG-24 * [50]	270	Epirubicin and docetaxel	23.0%	nonsignificant further increase in pCR with trastuzumab (38.6% EDC vs. 26.5% ED)
	266	Epirubicin, docetaxel, and capecitabine	15.4%	
GeparSepto ** [51,52]	606	NAB-paclitaxel followed by EC	38.4% (35.0–42.0%)	4-year OS: 89.7%, iDFS: 84.0%, DDFS: 85.6%
	600	sb-paclitaxel followed by EC	29.0% (25.0–33.0%)	4-year OS: 87.2%, iDFS: 76.3%, DDFS: 81.0%
GeparQuinto [53,54]	308	EC followed by docetaxel + lapatinib	22.7%	3-year OS: 93.6% (89.9–96.0%) 3-year DFS: 83.7% (78.7–87.6%) 3-year DDFS: 87.0% (82.4–90.5%)
	307	EC followed by docetaxel + trastuzumab	30.3%	3-year OS: 91.7% (87.6–94.4%) 3-year DFS: 84.8% (80.0–88.5%) 3-year DDFS: 86.2% (81.5–89.7%)
TRAIN-2 [55]	212	FEC followed by paclitaxel and carboplatin + trastuzumab and pertuzumab concurrently with all chemotherapy cycles	67% (60.0–73.0%)	
	206	Paclitaxel and carboplatin + trastuzumab and pertuzumab concurrently with all chemotherapy cycles	68% (61.0–74.0%)	
GeparQuattro *** [56,57]	471	EC then docetaxel	22.3%	
	471	EC then docetaxel + capecitabine	19.5%	
	479	EC then docetaxel followed by capecitabine	22.3%	

* Patients with HER2-positive disease were further randomized to receive trastuzumab or not. ** Patients with HER2-positive disease received dual antibody treatment with trastuzumab and pertuzumab. *** Patients with HER2+ tumors received trastuzumab concomitantly with all cycles. AC: doxorubicin and cyclophosphamide; ALNs: axillary lymph nodes; AT: doxorubicin and paclitaxel; CMF: cyclophosphamide, methotrexate, and fluorouracil; DDFS: distant disease-free survival; DFS: disease-free survival; EC: epirubicin and cyclophosphamide; ED: epirubicin and docetaxel; EDC: epirubicin, docetaxel, and capecitabine; EFS: event-free survival; FEC: fluorouracil, epirubicin, and cyclophosphamide; iDFS: invasive disease-free survival; OS: overall survival; pCR: pathological complete response.

**Table 2:** Key publications highlighting predictive and prognostic biomarkers in early breast cancer in the neoadjuvant setting.

Reference	Type of Publication (Number of Studies)	N	Breast Cancer Subtypes	Biomarkers Studied	Highlights
Chen et al. [77]	Systematic review and meta-analysis (43)	11,099	HER2-positive	*PIK3CA* mutation status	*PIK3CA* mutation was significantly associated with a lower pCR rate (OR = 0.23, 95% CI, 0.19–0.27, *p* < 0.001). This association remained significant irrespective of the type of anti-HER2 therapy (single-agent or dual-agent) and HR status. There were no significant differences in DFS between *PIK3CA* mutated and wild-type disease.
De Moraes et al. [78]	Systematic review and meta-analysis (29)	6161	TNBC	TILs	Compared with the low-TIL expression group, higher TIL infiltrations were associated with significant improvement in DFS (HR 0.71, 95% CI, 0.61–0.82; *p* < 0.00001) and OS (HR 0.76, 95% CI, 0.63–0.90; *p* = 0.002) rates. Improved pCR rates for the higher TIL group than control for both the TIL (OR 1.29, 95% CI, 1.13–1.48; *p* = 0.0003) and Ki-67 (OR 2.74, 95% CI, 2.01–3.73; *p* < 0.00001) analyses.
Myers et al. [79]	Large single-institution retrospective cohort (1)	1426	HER2-positive, TNBC, HR-positive/HER2-negative	*BRCA1/2* mutation status	pCR was achieved in 42% of *BRCA1* carriers, 21% of *BRCA2* carriers, and 26% of noncarriers (*p* = 0.001). Among patients with cN+ disease, nodal pCR was more frequent in *BRCA1/2* carriers compared to noncarriers (55% vs. 43%, *p* = 0.015); this was seen in HR+/HER2− and TNBC subtypes. *BRCA1* status and TNBC and HER2+ subtypes were independently associated with pCR.
Nader-Marta et al. [80]	Systematic review and meta-analysis (57)	5779	HER2-positive, TNBC, HR-positive/HER2-negative	ctDNA	ctDNA detection was associated with worse DFS at baseline (HR 2.98, 95% CI, 1.92–4.63), after NACT (HR 7.69, 95% CI, 4.83–12.24), and during follow-up (HR 14.04, 95% CI, 7.55–26.11). ctDNA detection at all timepoints was associated with worse OS (at baseline: HR 2.76, 95% CI, 1.60–4.77; after NACT: HR 2.72, 95% CI, 1.44–5.14; and during follow-up: HR 9.19, 95% CI, 3.26–25.90).
Papakonstantinou et al. [81]	Systematic review and meta-analysis (11)	653	HER2-positive, TNBC, HR-positive/HER2-negative	ctDNA	ctDNA detection at baseline and after completion of NACT was significantly associated with worse RFS (HR 4.22, 95% CI, 1.29–13.82 and HR 5.67, 95% CI, 2.73–11.75, respectively) and worse OS (HR 19.1, 95% CI, 6.9–53.04 and HR 4.00, 95% CI, 1.90–8.42, respectively). ctDNA was not associated with the probability of achieving a pCR.
Pohl-Rescigno et al. [82]	Secondary analysis of GeparOcto trial (1)	914	HER2-positive, TNBC, HR-positive/HER2-negative	*BRCA1/2* mutation status	Higher pCR rates were observed in patients with *BRCA1/2* variants than in patients without (60.4% vs. 46.7%; OR, 1.74, 95% CI, 1.13–2.68; *p* = 0.01). Variants in non-*BRCA1/2* breast cancer predisposition genes were not associated with therapy response. Patients with TNBC with *BRCA1/2* variants achieved the highest pCR rates in both the PMCb arm (74.3% vs. 47.0% without *BRCA1/2* variant; OR, 3.26, 95% CI, 1.44–7.39; *p* = 0.005) and the iddEPC arm (64.7% vs. 45.0%; OR, 2.24, 95% CI, 1.04–4.84; *p* = 0.04). A positive *BRCA1/2* variant status was also associated with elevated pCR rates in patients with HR-positive/HER2-negative cancer (31.8% vs. 11.9% without *BRCA1/2* variant; OR, 3.44, 95% CI, 1.22–9.72; *p* = 0.02).

CI: confidence interval; ctDNA: circulating tumor DNA; DFS: disease-free survival; HR: hazards ratio/hormone receptor; HER2: human epidermal growth factor-2; iddEPC: intense dose-dense epirubicin, paclitaxel and cyclophosphamide; NACT: neoadjuvant chemotherapy; PMCb: paclitaxel/non-pegylated liposomal doxorubicin and carboplatin; OR: odds ratio; OS: overall survival; pCR: pathological complete response; RFS: relapse-free survival; TILs: tumor-infiltrating lymphocytes; TNBC: triple-negative breast cancer.

**Table 3:** Clinical trials investigating the efficacy of immunotherapy in the neoadjuvant setting for early-stage TNBC.

Trial	N	Intervention	Control	Treatment Post Surgery	Results
KEYNOTE-522 [110,111] (NCT03036488)	1174	Pembrolizumab every 3 weeks plus paclitaxel and carboplatin for four cycles, followed by four cycles of pembrolizumab plus AC/EC every 3 weeks for 12 weeks	Placebo every 3 weeks plus paclitaxel and carboplatin for four cycles, followed by four cycles of placebo plus AC/EC every 3 weeks for 12 weeks	Pembrolizumab or placebo every 3 weeks for up to 9 cycles	Significant increase in pCR rate (63.0% vs. 55.6%) Significant increase in EFS (84.5% vs. 76.8%)
NeoTRIP Michelangelo [112] (NCT002620280)	280	Carboplatin AUC 2 and nab-paclitaxel on day 1 and day 8 every 3 weeks with atezolizumab on day 1 every 3 weeks for eight cycles	Carboplatin AUC 2 and nab-paclitaxel on day 1 and day 8 every 3 weeks for eight cycles	4 cycles of an anthracycline regimen as per investigator’s choice	Nonsignificant increase in pCR rate (48.6% vs. 44.4%)
GeparNuevo [93,113] (NCT02685059)	174	Durvalumab every 4 weeks added to nab-paclitaxel weekly for 12 weeks, followed by durvalumab every 4 weeks plus EC every 2 weeks	Placebo every 4 weeks added to nab-paclitaxel weekly for 12 weeks, followed by placebo every 4 weeks plus EC every 2 weeks	According to standard of care (not part of the study)	Nonsignificant increase in pCR rate (53.4% vs. 44.2%) Significant gains in survival: 3-year iDFS (85.6% vs. 77.2%) 3-year DDFS (91.7% vs. 78.4%) 3-year OS (95.2% vs. 83.5%)
IMpassion031 [94] (NCT03197935)	333	Atezolizumab every 2 weeks added to nab-paclitaxel weekly for 12 weeks, followed by atezolizumab every 2 weeks plus AC every 2 weeks for four cycles	Placebo every 2 weeks added to nab-paclitaxel weekly for 12 weeks, followed by placebo every 2 weeks plus AC every 2 weeks for four cycles	Intervention arm: atezolizumab every 3 weeks for 11 cycles Control arm: monitoring for up to 1 year after start of therapy	Significant increase in pCR rate (58% vs. 41%) *
I-SPY2 [114] (NCT01042379)	29 (TNBC) 180 (control)	Pembrolizumab every 3 weeks for four cycles concurrently with weekly paclitaxel for 12 weeks, followed by AC every 2–3 weeks for four cycles	Weekly paclitaxel for 12 weeks, followed by AC every 2–3 weeks for four cycles	According to standard of care (not part of the study)	Increase in pCR rate (60% vs. 22%)

* The trial was not powered for long-term survival outcomes (EFS and OS). AC: doxorubicin and cyclophosphamide; AUC: area under the curve; DDFS: distant disease-free survival; EC: epirubicin and cyclophosphamide; EFS: event-free survival; iDFS: invasive disease-free survival; OS: overall survival; pCR: pathological complete response; TNBC: triple-negative breast cancer.
